# Comparison of phosphorylation patterns across eukaryotes by discriminative N-gram analysis

**DOI:** 10.1186/s12859-015-0657-2

**Published:** 2015-07-30

**Authors:** Itziar Frades, Svante Resjö, Erik Andreasson

**Affiliations:** Department of Plant Protection Biology, Swedish University of Agricultural Sciences, Alnarp, SE-230 53 Sweden

**Keywords:** Comparative phosphoproteomics, Evolutionary phosphoproteomics, N-gram analysis

## Abstract

**Background:**

How protein phosphorylation relates to kingdom/phylum divergence is largely unknown and the amino acid residues surrounding the phosphorylation site have profound importance on protein kinase–substrate interactions. Standard motif analysis is not adequate for large scale comparative analysis because each phophopeptide is assigned to a unique motif and perform poorly with the unbalanced nature of the input datasets.

**Results:**

First the discriminative n-grams of five species from five different kingdom/phyla were identified. A signature with 5540 discriminative n-grams that could be found in other species from the same kingdoms/phyla was created. Using a test data set, the ability of the signature to classify species in their corresponding kingdom/phylum was confirmed using classification methods. Lastly, ortholog proteins among proteins with n-grams were identified in order to determine to what degree was the identity of the detected n-grams a property of phosphosites rather than a consequence of species-specific or kingdom/phylum-specific protein inventory. The motifs were grouped in clusters of equal physico-chemical nature and their distribution was similar between species in the same kingdom/phylum while clear differences were found among species of different kingdom/phylum. For example, the animal-specific top discriminative n-grams contained many basic amino acids and the plant-specific motifs were mainly acidic. Secondary structure prediction methods show that the discriminative n-grams in the majority of the cases lack from a regular secondary structure as on average they had 88 % of random coil compared to 66 % found in the phosphoproteins they were derived from.

**Conclusions:**

The discriminative n-grams were able to classify organisms in their corresponding kingdom/phylum, they show different patterns among species of different kingdom/phylum and these regions can contribute to evolutionary divergence as they are in disordered regions that can evolve rapidly. The differences found possibly reflect group-specific differences in the kinomes of the different groups of species.

**Electronic supplementary material:**

The online version of this article (doi:10.1186/s12859-015-0657-2) contains supplementary material, which is available to authorized users.

## Background

Post-translational modifications offer ways to quickly and reversibly regulate protein activity, subcellular localization and stability. One of the most abundant modification is protein phosphorylation and dephosphorylation, catalyzed by kinases and phosphatases, respectively. There are thousands of distinct phosphorylation sites in a given cell and 30 % of proteins encoded in a genome can be phosphorylated [1, 2]. Phosphoregulation has great potential to contribute to the evolution of phenotypic diversity though mutations in phosphorylation sites that can create new crosstalk in signaling networks and regulate the activity of proteins that were once constitutively active [3]. The contribution of these protein modifications to evolutionary divergence and convergence is still largely unknown.

The majority of phosphorylation sites are in disordered regions of proteins that can have a rapid evolution due to their lack of structural constraints [4, 5]. In contrast to structural domains in proteins, phosphorylation sites are short disordered motifs that specify interactions in regulatory networks and they should be able to arise rapidly from random sequences [6, 7]. These interactions can be modified by point mutations or insertions and deletions or gene duplication.

Boekhorst *et al.* compared phosphoproteomics datasets of five eukaryotes and found a high overlap between closely related species (700 sites for human and mouse), in contrast with a single site for fish and yeast [8]. They identified homologous phosphosites by using the Smith-Waterman algorithm to do an all-against-all similarity search of all full-length phosphoproteins. Freschi *et al.* [9] studied the evolution of mammalian phosphoregulation by comparing human and mouse phosphoproteomes. They found that many of the positions that are phosphorylated in human and mouse were conserved at the residue level and these conserved sites were phosphorylated in both species 2.5 times more often than expected by chance alone. These results support the hypothesis that the evolutionary turnover of phosphorylation sites contributes to the divergence in phosphorylation profiles. They also found that these sites tend to be phosphorylated by the same kinases, which means that phosphoregulation was preserved. Cross-species comparative studies of genetic interactions performed by Beltrao *et al.* [10] revealed that kinases show a faster than average rate of functional divergence. Van Wijk *et al.* [11] used 27 published and unpublished in-house mass spectrometry–based phosphoproteome data sets for *Arabidopsis thaliana *and performed an assembly of 60,366 phosphopeptides matching to 8141 non-redundant proteins. Then, they determined the distribution of phosphoproteins across functions and subcellular locations, and identified phosphomotifs for different subcellular locations using motif-x and MMFPh motif finders.

Recent high-throughput phosphoproteomics studies have resulted in an accumulation of phosphopeptide datasets for many species. Analysis of phosphopeptides is usually done by defining phosphorylation motifs, which are short distinct local amino acid patterns surrounding the local phosphorylation site shared by different proteins that are highly frequent in an species, tissue or treatment. Problems are that in standard motif identification methods, such as motif-x and MMFPh, each phophopeptide is assigned to a unique motif and they perform poorly with unbalanced input datasets. This calls for use of alternative methods to perform a large scale comparative analysis. The term n-gram or n-mer typically refers to all the possible substrings, of length n, that are contained in a string; therefore an n-gram is a contiguous sequence of n items from a given sequence. It is possible to build classification models from sequences using the statistical properties of n-grams. In this case, we have defined discriminative n-grams as contiguous short peptide sequences of n items derived from the phosphoproteins that are highly frequent in one species but are either minimally present or absent in other species. N-grams, have been used to align DNA sequences [12], clustering sequences [13], predicting subcellular localization [14] and for functional annotation of protein sequences [15], but not to identify kingdom/phylum-specific phosphorylation motifs. In protein sequence classification, the objective is to identify the sequence elements that can discriminate between classes. Identification of discriminative phosphopeptide motifs or phophopeptide *n*-grams that can precisely discriminate between species is a classification problem itself. Ganapathiraju et al. [16, 17] using selective n-grams performed optimized protein-family classifications by training Bayesian classifiers and neural networks. In other studies, the distribution of n-grams have served as a proteome-signature for species determining evolutionary divergence at the genus level [18].

In this study we performed a novel comparative analysis of phosphorylation events with serine type phosphosites between different species of eukaryotes. To achieve this, we performed a discriminative n-gram based analysis to identify kingdom/phylum-specific phosphorylation motifs. First the discriminative n-grams of five species from five different kingdoms/phyla were identified and from the enriched counts of these n-grams in each species the normalized frequencies discriminating the species were derived. Then a signature with the discriminative n-grams that could be found in other species from the same kingdoms/phyla was created. A testing dataset with five other species in the same kingdoms/phylum was used to validate the potential of the normalized frequencies of the discriminative n-grams in the signature to classify the species in their corresponding kingdom/phylum.

The problem of PTM site prediction is traditionally an issue of false-positive over-prediction. Predictions for post-translational modifications reduce the false-positives if they are frequently observed in a protein family as opposed to a single protein sequence [19, 20] and it is dangerous to build predictors without considering the physico-chemical properties used to create sequence families. A comparison of phosphorylation discriminative n-grams in different species was done by grouping these motifs in clusters according to physico-chemical properties and analyzed whether the differences in the distribution of these clusters between the different species could be used to discriminate kingdoms/phyla. The distribution between the different species of hydrophobic, negative, positive and proline amino acids along the phosphorylation sites and the surrounding sequences holding the discriminative n-grams was also analyzed.

We believe that this study creates a basis for identifying kingdom/phylum specific phosphorylation substrates of protein kinases for kinase inhibitor based drugs and pesticides.

## Methods

### Datasets

To identify motifs with Motif-x and MMFPh *Phytophthora infestans* [21], *Arabidopsis thaliana*, *Saccharomyces cerevisiae* and *Homo sapiens* phosphoproteomics datasets were used (Table 1). For the discriminative n-gram analysis, a training dataset composed of ten phosphoproteomics datasets was used (two from five different species) (Table 1). For the test set generation, five other datasets from five different species from the same kingdom/phylum in the training set were used (Table 1).

**Table 1:** Serine centered phosphopetide sequences of 21 length, n-grams of varying size (6 to 21 mer) and references from the datasets in each kingdom/phylum and species under study in the training set and the test set. For each species in the training set two datasets were used, and hence, two numbers are given. There were many more n-grams than phospho-sites, due to the window of phospho-sites (21) and varying length of n-grams within the sites

Training set	Number of phosphopeptides	Number of n-grams	References	Kingdom	Phylum
*Arabidopsis thaliana*	2903 and 4270	349724 and 527397	[40, 41]	Plantae	
*Homo sapiens*	1972 and 4075	200661 and 454563	[50, 51]	Animalia	Chordata
*Drosophila melanogaster*	6363 and 6362	671933 and 596922	[52, 53]	Animalia	Arthropoda
*Saccharomyces cerevisiae*	6343 and 1178	712345 and 116095	[54, 55]	Fungi	Ascomycota
*Plasmodium falciparum*	744 and 1048	93799 and 137899	[56, 57]	Chromalveolata	Apicomplexa
*Oryza sativa*	447	50007	[58]	Plantae	
*Mus musculus*	7372	811443	[59, 60]	Animalia	Chordata
*Caenorhabditis elegans*	4003	436055	[61]	Animalia	Nematoda
*Schizosaccharomyces pombe*	1362	155639	[62]	Fungi	Ascomycota
*Toxoplasma gondii*	1388	172714	[56]	Chromalveolata	Apicomplexa

### Identification of motifs

#### Motif-x and MMFPh

Motif-x [22, 23] and MMFPh [24] were used with pre-aligned phosphosites from different phosphoproteomics datasets to get the significant phosphorylation motifs. Both methods iteratively extract overrepresented motifs from pre-aligned peptides through comparison with a dynamic statistical background. Both employ a local assessment of individual amino acid/position pairs during construction of a motif, but Motif-x performs a greedy growing, that is, makes locally the optimal choice at each iteration, while MMFPh considers all the possible multiple ways to grow to a motif from more than one fixed position at each iteration (e.g. S → PxS → PxSR or S → SR → PxSR), guaranteeing to find all significant maximal motifs. These methods use the binomial probability as a scoring system and this is dependent on foreground matches, foreground size, background matches and background size. The number of motifs found depends on the significance threshold and the minimum number of occurrences necessary to consider a given motif significant. A Friedman test was used to measure whether there were significant differences between the scores produced by Motif-x and MMFPh. The number of motifs shared by the two methods as well as the exclusive ones from each method were also compared. Similarly, the difference between the scores produced using 21 or 13-mer peptides was measured, and the motifs generated by using the different lengths were compared. The motifs in each species were aligned and the uniquely significantly enriched motifs in *P. infestans* compared to *A. thaliana*, *S. cerevisiae* and *H. sapiens* were identified. One way ANOVA was also used to test whether there were motifs with a significant higher score in *P. infestans*.

#### 21 and 13-mer centered phospho-serine pseudoalignment peptide sequences

In kinase-substrate interactions, a phosphosite containing the peptide sequence that includes the surrounding specificity-determining residues fits into a kinase active site [25]. The specificity for kinases is dictated by both the amino acid sequence motif surrounding the phosphorylated residues and the three-dimensional structure of the substrate proteins [26].

Several methods use the surrounding region of −6 to +6 amino acids in order to display motifs [27, 28]. Others use a length of each extracted peptide of 21 with a measured phosphorylated residue in the 11th position [29].

21 and 13-mer phospho-serine centered pseudoalignment peptide sequences were used with Motif-x and MMFPh to extract motifs that hold the kinase specificity-determining residues, while only 21-mer phospho-serine centered pseudoalignment peptide sequences were employed for generating the n-grams.

#### Detection of discriminative motifs: n-grams based algorithm

The n-gram approach described in [15] was used to construct a phosphoproteome-signature composed by n-grams distinguishing the phosphoproteome of various species belonging to different kingdoms/phyla. To achieve this the discriminative n-grams from a training set of serine centered phosphopetides belonging to 10 datasets (two from each species) were computed and their normalized frequencies recorded: first, n-grams of varying size (6 to 21 mer) were extracted from each dataset’s serine centered phosphopetide sequences of 21 length using the ‘tau’ r package. Second, their frequency counts were summed to obtain the enriched counts. Third, a dampening factor, which gives more weight to n-grams that appear in fewer species and vice-versa, was used to normalize the weights of n-grams from different unbalanced phosphorylation datasets and generate the normalized frequencies of n-grams. Finally, a discriminative ratio was calculated for each n-gram to identify the species that contained this n-gram with a frequency at least T times higher than the average frequency of the second and third highest frequencies having species. In parallel the n-grams and their normalized frequencies were computed on a testing dataset with other five different species, each of which had a species in the training dataset belonging to the same kingdom/phylum. The validation set was created by selecting the same n-grams in the training set and calculating the enriched counts and the normalized frequencies.

#### Phosphoproteome-signature: detection and evaluation of Kingdom/phylum specific motifs

The discriminative n-grams are designed to discriminate between the species in the training set only. A phosphoproteome-signature was generated holding a subset of discriminative n-grams that are kingdom specific. The discriminative n-grams for each species in the training dataset that were present in at least the species of the same kingdom/phylum in the testing dataset were included in the signature. It was evaluated the signature’s capability to distinguish the phosphoproteome of various kingdoms/phyla using the normalized frequencies derived from enriched counts of n-grams among the phosphopetides found in the different species. This was carried out by using John Platt’s sequential minimal optimization algorithm for training a support vector classifier with the normalized polynomial kernel on the normalized frequencies of the discriminative n-grams with the species in the training dataset [30]. Then it was evaluated the performance of the discriminative n-grams’ normalized frequencies in the signature to classify each species in the testing set with the species in the training set belonging to the same kingdom/phylum.

Additionally, we performed a hierarchical cluster analysis using pvclust R package [31] in order to explore the signature’s capability to classify each species in the testing set with the species in the training set using unsupervised classification methods. Bootstrap resampling techniques were used to assess the uncertainty in hierarchical cluster analysis by calculating probability values (p-values) for each cluster in the dendrogram that represents the possibility that the cluster is the true cluster. Two types of p-values were available: bootstrap probability (BP) value and approximately unbiased (AU) p-value.

### Analysis of orthologs among the proteins with discriminative n-grams

By analysis of ortohlogs we distinguished between the motifs which identity was a property of phosphosites and motifs that were derived from the species-specific or kingdom/phylum specific protein inventory. We used the Homologene [32] to determine if the proteins with kingdom/phylum specific discriminative n-grams in each species had orthologs in other species. We made three calculations: (1) The proportion of proteins having kingdom/phylum specific discriminative n-grams with no orthologs in other species, (2) The proportion of proteins with kingdom/phylum specific discriminative n-grams with orthologs only in a species of the same kingdom/phylum, and (3) The proportion of proteins with discriminative n-grams with orthologs in other kingdoms/phyla.

### Distribution of clusters of discriminative motifs

The discriminative n-grams were mapped back to the their corresponding phosphopeptides and the values of each amino acid in each position in the 21-mer sequences were substituted with binary physico-chemical properties defined by [33]. From this data hydrophobicity, negative, positive and proline content were analyzed. Then, for each physico-chemical property the average values of the phosphopeptide sequences belonging to each discriminative n-gram were calculated.

For each physico-chemical property k-means (k = 10) partitional clustering algorithm was used to cluster the n-grams according to their average values. Consensus or ensemble clustering is a way of reconciling clustering information about the same dataset coming from different sources. It refers to the situation in which a number of different (input) clustering results have been obtained for a particular dataset and goal is to find a single (consensus) clustering. In this case the procedure was computed to create a consensus cluster of each cluster of the individual physico-chemical properties using soft least squares Euclidean consensus partition to cluster the motifs according to all the physico-chemical properties together. The R packages “cluster” and “clue” were used to implement the methodology. This resulted in grouping the discriminative motifs in clusters of motifs of similar physico-chemical nature.

### Functional analysis

For each of the 10 species in the training and testing set, the n-grams were mapped onto their original serine centered phosphopeptides and these were mapped again into their corresponding protein sequence (discriminative proteins). The discriminative proteins belonging to each of the 10 species were used to identify significantly enriched KEGG pathways by means of hypergeometric test using the KEGG Orthology Based Annotation System [34] except for the mice data, for which WebGestalt [35, 36] was used. Then, it was measured whether these discriminative proteins were functionally conserved between each species in the training set and their corresponding species of the same kingdom/phylum in the test set as well as whether they were different between species from different kingdoms/phyla.

### Top discriminative n-gram logos

For each species the top n-grams that have the greater discriminative ratio were extracted from the signature. The top discriminative n-gram logos were generated using WebLogo [37] from the serine centered phosphopeptides that map each of the top n-grams. In each species the top n-grams matching phosphopeptides that had the highest discriminative ratio were used to create the logos. The discriminative ratio calculated for each n-gram identifies how many times higher is the frequency of the n-gram in the species having the highest frequency than the average frequency in the species that have second and third highest frequencies. This means that the n-grams having highest discriminative ratios will be the ones having greater differences in frequency among the different species in the training set. As the different species had a different highest discriminative ratio, distinct discriminative ratio thresholds were defined to select the top n-grams to create the top discriminative n-gram logos in each species. The criterion to assess a cutoff was defined on the basis of getting from each species the higher discriminative ratios at which the phosphopeptide sequences showed clear regularities. This allowed comparing the amino acid composition of the top n-grams in each species.

### Secondary structure prediction

We used PSIPRED to predict the secondary structure (beta sheets, alpha helices and coils) from the primary sequence of the proteins holding the kingdom/phylum specific discriminative n-grams from all the species in the signature. We recorded the proportion of random coils in all the proteins and in the serine centered 21 and 13 mer phosphopeptides of each species. The random coil is not a true secondary structure, but is the class of conformations that indicate an absence of regular secondary structure that can be thought as a disordered region.

## Results and discussion

### Pitfalls in the motif detection by conventional methods

We investigated whether there were significant differences between the scores produced by the two most commonly used motif generators, Motif-x and MMFPh, by analyzing our recently published *P. infestans* phosphopeptide dataset [21]. By Friedman test, significant differences between both methods were found among the binomial probabilities of the two methods (p = 0.01279). This difference is probably attributed to different ways of growing the motifs: Motif-x performs a greedy growing while MMFPh considers all the possible extensions at each iteration from the multiple ways to grow a motif from more than one fixed position. Little overlap in the motifs detected by the two methods was found (Additional file 1: Table S1). In contrast, no statistical difference was found between scores produced using 21 or 13-mer serine centered pseudoalignment peptide sequences to generate the motifs (p = 0.715). The motifs overlap and exclusivity between the two serine centered phosphopeptide lengths were analysed, and we found a greater number of motifs detected exclusively using 21 mer than using 13 mer (Additional file 1: Table S1).

The motifs in *P. infestans* , *A. thaliana*, *S. cerevisiae* and *H. sapiens* (Additional file 2: Table S2) from both methods and phosphopeptide lengths were extracted and aligned. Among these motifs, 24 were found to be unique in *P. infestans* compared to the other species and were sorted by motif score (Additional file 1: Table S3). In this analysis the PxSPR motif was the uniquely enriched significant motif in *P. infestans* with the highest score even though this motif is known to be abundant in MAP kinase signaling in *A. thaliana* [38, 39]. The SPR motif also had a significantly higher score in *P. infestans* (Additional file 1: Table S3) than *A. thaliana*, *S. cerevisiae* and *H. sapiens,* even though it is a common motif in all the investigated species.

Thus, Motif-x and MMFPh failed to find species specific motifs that can be used for phosphoprotein classification and to find discriminative motifs. The most obvious explanation for this result is that when these methods find a significant motif shared by some sequences, these are not used again to find a new motif and therefore sequences can be grouped to form the wrong motif and interesting motifs are missed. An additional problem that these methods are unable to deal with unbalanced datasets as in these methods it is decided whether a motif is significant when the motif is present in at least a pre-specified number of phosphopeptide sequences. To test this, we used three individual phosphosite detection experiments in *A. thailiana* [40, 41] and the number of different motifs in each individual dataset were computed. Additionally motifs were computed by grouping the phosphosites from the three experiments and we found 61 more motifs than the number of motifs generated by summing the motifs determined individually in each experiment (Table 2). In summary, these results call for alternative methods to compare phosphoproteomic datasets.

**Table 2:** Motif analysis of three individual phosphosite detection experiments in *A. thailiana*. The number of different motifs in each of the three individual experiment was computed and the resulting three numbers of motifs were summed (Sum of number of motifs from individual experiments). Additionally the motifs that are obtained by grouping the phosphosites from the three experiments (Sum of experiments) were computed

Dataset: *A. thaliana* PhosPhAt 4.0 [40, 41]	Number of motifs	Number of phosphosites	Minimum number of occurences
Experiment1	116	1733	5
Experiment2	3	178	5
Experiment3	99	6862	21
Sum of number of motifs from individual experiments	182	8773	5;5;21
Sum of experiments	243	8773	27

### Kingdom/phylum specific phosphorylation patterns

In order to identify kingdom/phylum specific phosphorylation patterns, n-grams of varying size (6 to 21 mer) were extracted from serine centered phosphopeptide sequences of 21-mer length in each dataset in the training and testing sets (Table 1). N-grams that had a discriminative ratio greater than 0.05 were considered as discriminative. The characteristic frequency of amino acids (kingdom/phylum specific discriminative n-grams) in the phosphopeptide sequences of each kingdom/phylum is referred as a phosphoproteome-signature. A phosphoproteome-signature with 5540 kingdom/phylum specific discriminative n-grams was obtained by finding the discriminative n-grams from each species in the training dataset that also were present among the n-grams from a species of the same kingdom/phylum in the testing dataset (Additional file 3: Table S4). This method has also been descried as suitable to compare unbalanced datasets [15].

**Fig. 1:**
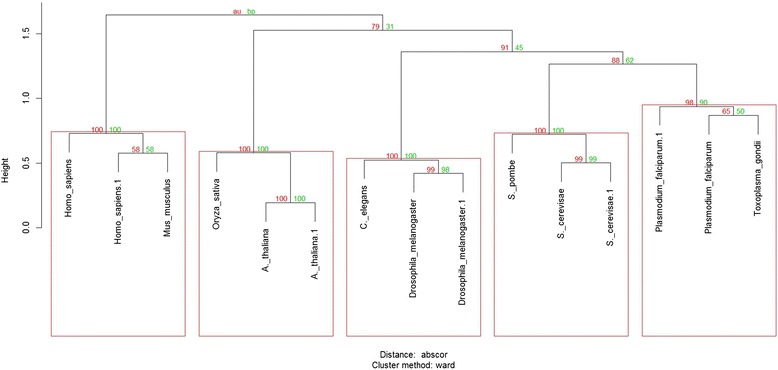
Hierarchical cluster analysis of the normalized frequencies of the discriminative n-grams present in each speciesThe dendogram shows that different species having the same kingdom/phylum cluster together

The normalized frequencies of the discriminative n-grams in this signature were able to classify each of the species in the test set as belonging to the same group as a species from the same kingdom/phylum in the training set using a classifier for building support vector classification models or using hierarchical clustering (Table 3; Fig. 1). This way the kingdom/phylum-specific phosphorylation patterns were defined by discriminative n-gram analysis. These might reflect an evolutionary divergence between kingdoms/phyla, and conservation within kingdoms/phyla of the protein phosphorylation in the studied species.

**Table 3:** Confusion matrix of the signature pairing equal kingdom/phylum species

*A. thaliana*	*H. sapiens*	*D. melanogaster*	*S. cerevisae*	*P. falciparum*	classified as
1	0	0	0	0	*O. sativa*
0	1	0	0	0	*M. musculus*
0	0	1	0	0	*C. elegans*
0	0	0	1	0	*S. pombe*
0	0	0	0	1	*T. gondii*

In comparative and integrative analysis of phosphoproteomes it can be difficult to know if phosphosites identified in only one sample are the result of species specific phosphorylation, or reflect missing data and biases that are introduced by different experimental workflows [42]. To minimize these problems our method identified phosphorylation motifs for each kingdom/phylum that were more abundant in the two datasets from an species in the training set, while these are also more abundant in the dataset of the corresponding species in the test set.

### Analysis of orthologs among the proteins with discriminative n-grams

Orthology analysis of the discriminative n-grams holding proteins showed that on average 77 % of these n-grams mapped to proteins having orthologs in different species and while the 23 % mapped to proteins that existed in the respective species only (Table 4 & Additional file 4: Table S5). The n-grams from the species in the training set share a great degree of orthology with the corresponding species in the testing set as they are conserved within each kingdom/phylum. More importantly, the proportion of discriminative proteins with n-grams having orthologs in other kingdoms/phyla shows that identity of the detected motifs is a property of phosphosites rather than a consequence of species-specific or kingdom/phylum specific protein inventory.

**Table 4:** Each species proportion of proteins having discriminative n-grams with no orthologs in other species, proteins having discriminative n-grams with orthologs only in a species of the same kingdom/phylum and proteins having discriminative n-grams with orthologs in other species

Species	Each species proportion of discriminative n-grams with no orthologs in other species	Each species proportion of discriminative n-grams with orthologs only in species of the same kingdom/phylum	Each species proportion of discriminative n-grams with orthologs outside the kingdom/phylum
*Arabidopsis thaliana*	24.4 %	63.1 %	12.5 %
*Oryza sativa*	0	58.2 %	41.8 %
*Homo sapiens*	3.6 %	87.1 %	9.3 %
*Mus musculus*	2.3 %	91.0 %	6.4 %
*Drosophila melanogaster*	40.0 %	5.4 %	54.6 %
*Caenorhabditis elegans*	22.5 %	11.7 %	65.8 %
*Saccharomyces cerevisiae*	62.3 %	15.9 %	21.7 %
*Schizosaccharomyces pombe*	33.3 %	21.2 %	45.4 %

### Grouping motifs according to physico-chemical properties

**Fig. 2:**
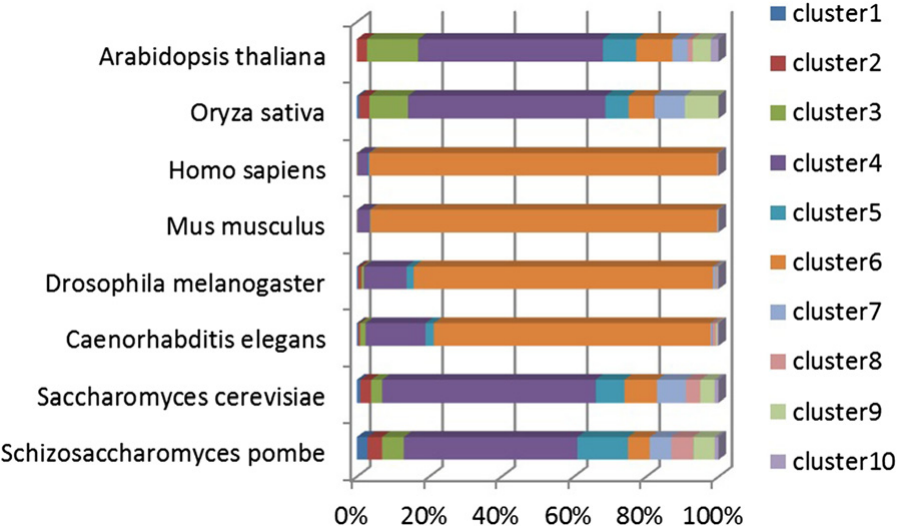
Distribution of clusters of discriminative motifs of similar physico-chemical nature among species

The n-grams of equal physico-chemical nature were grouped into clusters. The distribution of these clusters was almost the same between species in the same kingdom/phylum, meaning that the motifs in the clusters were similar (Additional file 1: Figure S1). There were clear distributional differences of the clusters between species of different kingdoms/phyla while the distribution of the species belonging to the same kingdom/phylums was similar (Fig. 2). Cluster four dominating in plants and fungi, was abundant in polar serines and acidic residues, while in the Animalia kingdom dominates cluster six holding serines, acidic and non-polar highly hydrophobic residues (Fig. 2 & Additional file 1: Figure S1). Within each cluster the species that belong to the same kingdom/phylum showed more similar patterns than the ones belonging to different kingdoms/phyla (Additional file 1: Figure S1).

**Fig. 3:**
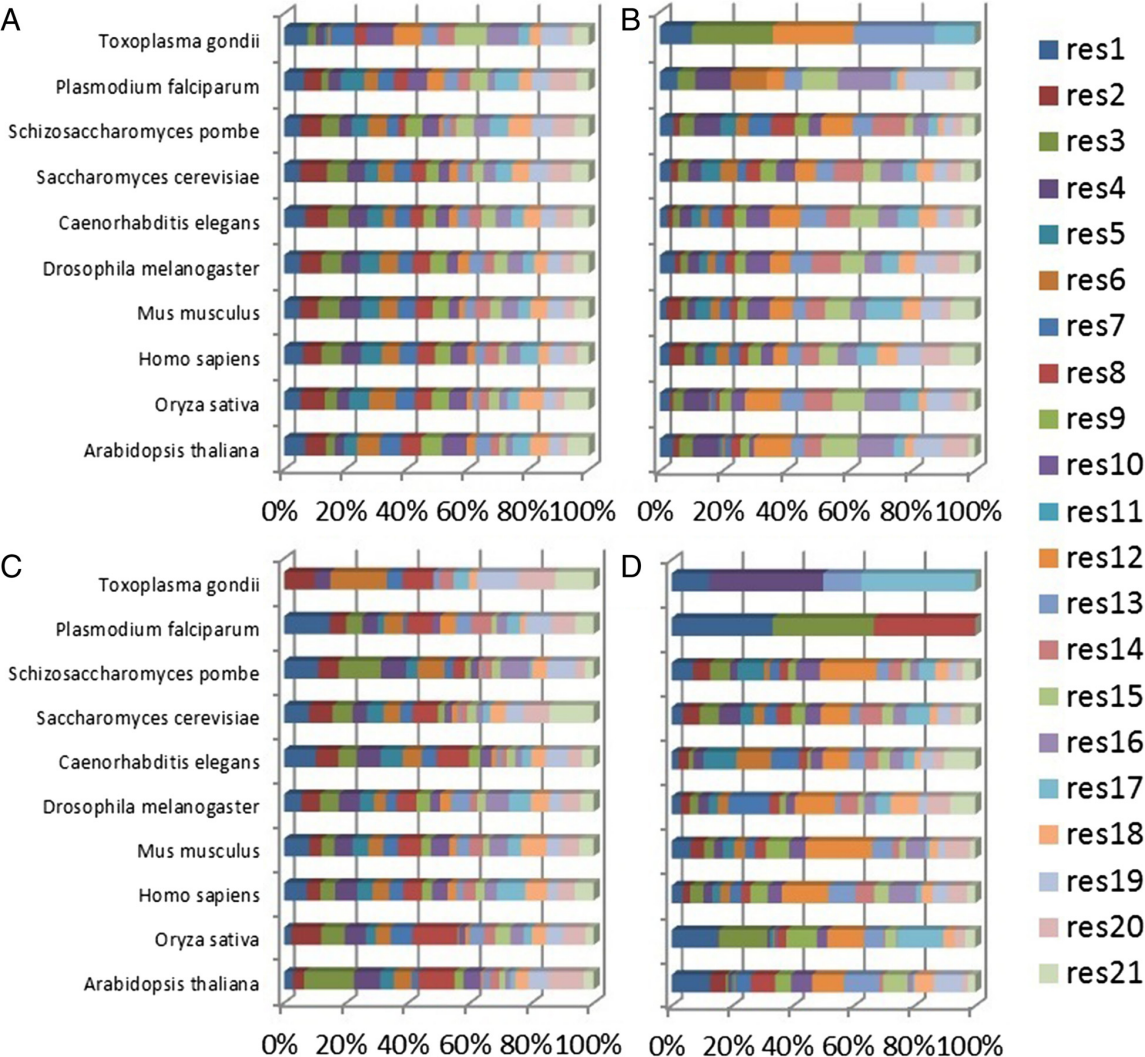
Proportion of hydrophobic (**a**), negative (**b**), positive (**c**) and proline (**d**) amino acids in the discriminative n-grams of each species

The proportion of hydrophobic, negative and positive amino acids as well as the proline content was analyzed within each cluster (Additional file 1: Figure S2). Results indicated that all clusters show a similar distribution of hydrophobic residues among the clusters while there are clear distributional differences for negative, positive and proline residues. This suggests that phosphorylation sites and the surrounding sequences are constrained in terms of hydrophobic patterns, probably due to the lack of structural preferences. The proportion of hydrophobic, negative and positive amino acids and the proline content in each species was also analyzed (Fig. 3). Again, the distribution of hydrophobic amino acids along the residues between the different species is similar, while for the rest of the studied physico-chemical properties there are greater differences in the distribution, indicating that the constraints in the hydrophobicity of phosphorylation sites and the surrounding sequences are conserved during evolution.

### Functional conservation among species of the same kingdom/phylum

**Fig. 4:**
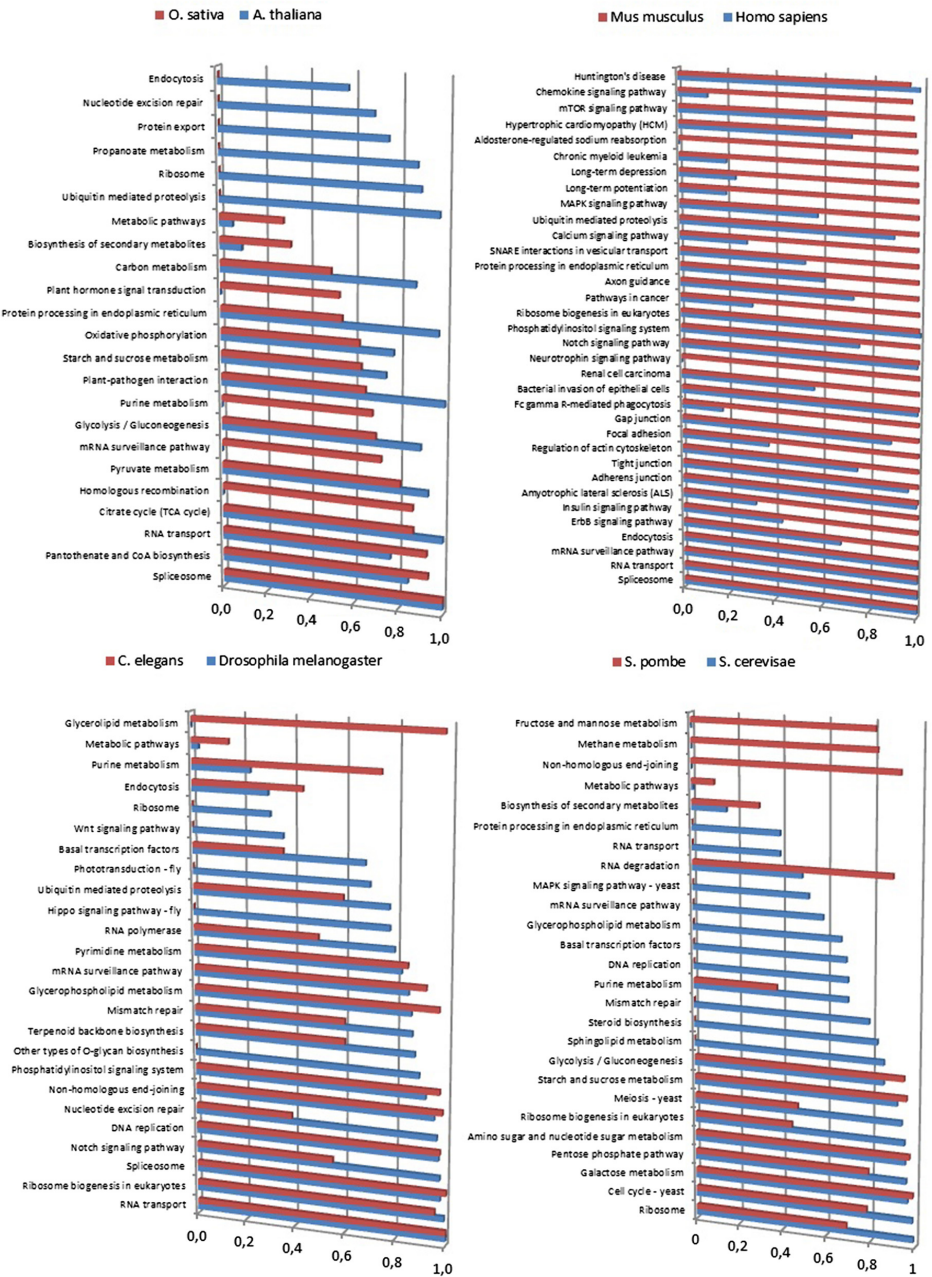
KEGG enrichment analysis of the phosphoproteins that match the discriminative n-grams. Functional conservation is found between the phosphoproteins that match the discriminative n-grams in closely related species belonging to the same kingdom/phylum. The histograms shows 1-p.value from the KEGG enrichment analysis

The discriminative n-grams in the signature were mapped to their corresponding proteins (Additional file 3: Table S4), generating lists of proteins containing discriminative n-grams for each group of species. The functional conservation of these proteins between each species in the training set and their corresponding species of the same kingdom/phylum in the test set was measured, as well as whether they had a diverse function among different kingdoms/phyla. The performed KEGG enrichment analysis of the proteins with discriminative n-grams showed that within each kingdom/phylum similar functions were conserved (Fig. 4); therefore the discriminative n-grams might be derived from conserved orthologous proteins or proteins with similar functions. We identified that within each kingdom in many cases orthologous proteins were responsible of enriching similar functions. Between kingdoms/phyla the functions were very dissimilar (Fig. 4). These results corroborate the fact that these proteins are kingdom/phylum specific and have the potential to discriminate the different kingdoms/phyla.

Among the discriminative proteins for humans and mice, there are many different signal transduction pathways, such as insulin, MAP kinase and calcium signaling pathways (Fig. 4). There are also a number of cytoskeletal proteins and proteins involved in cellular structure (Fig. 4). The insect discriminative proteins are dominated by enzymes involved in DNA- and RNA processing, and nucleotide metabolism (Fig. 4). The plant and fungal proteins containing the discriminative n-grams are more similar between them than to the Animalia kingdom (Fig. 4). Both are dominated by metabolic proteins, but there are also some other plant categories such as plant-pathogen interactions. The proteins from the plant discriminative n-grams exhibited a smaller number of functions (mostly central metabolic pathways such as glycolysis and the TCA cycle). On the other hand, the proteins from the fungal discriminative n-grams have a larger number of functions, including more specialized metabolic pathways such as sphingolipid metabolism and glycerophospholipid metabolism.

### Characterization of the top N-gram logos

**Fig. 5:**
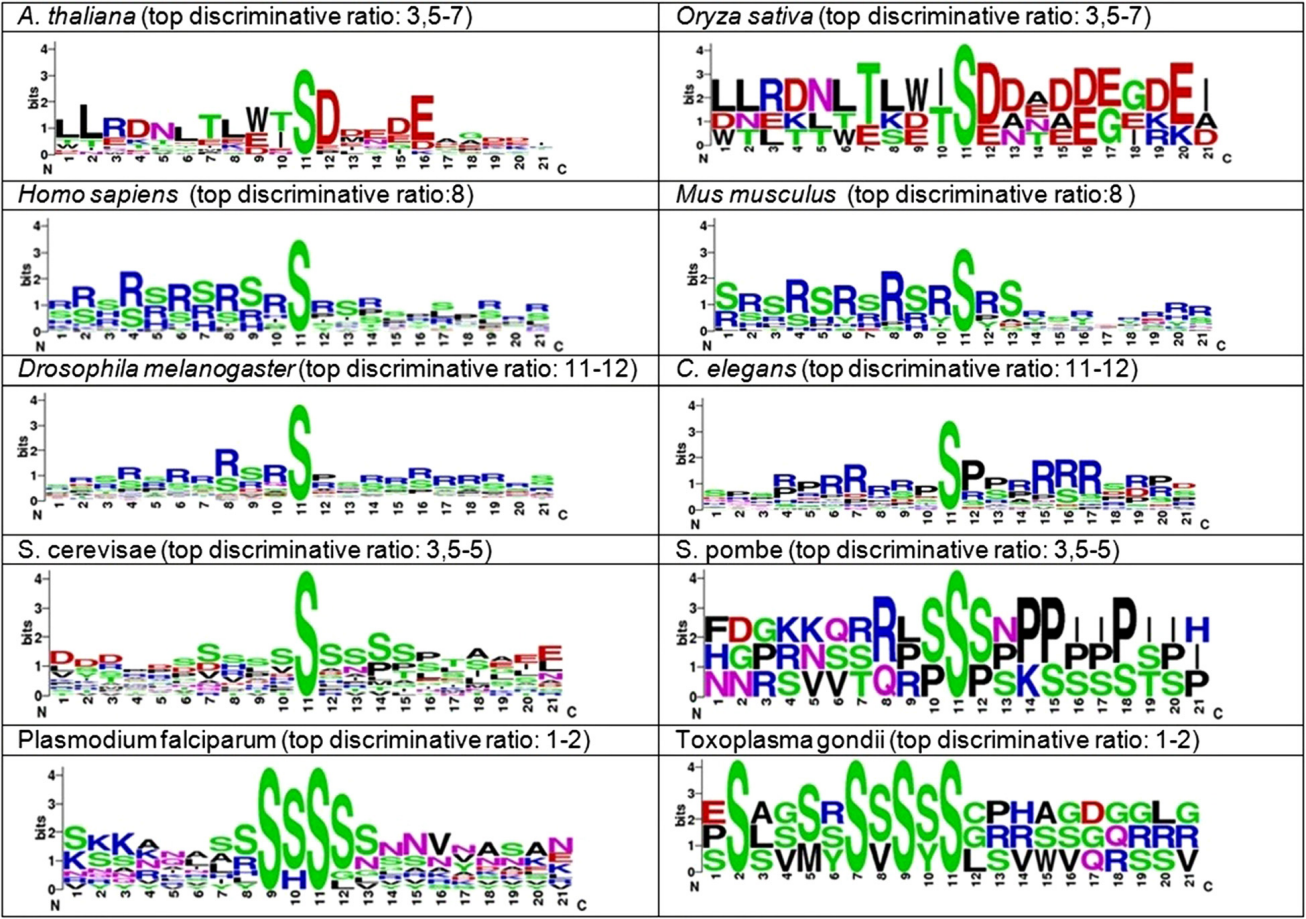
Logos of the top discriminative n-grams matching phosphopeptides having the maximum discriminative ratios in each species

The logos of the phosphopeptides derived from mapping back the top discriminative n-grams into the serine-centered phosphopeptides were generated (Fig. 5). The peptide sequences showed amino acid patterns that were conserved between each species in the training set and their corresponding species of same kingdom/phylum in the test set. These amino acid patterns were diverse among the different kingdom/phyla and they were able to classify the phosphorylation patterns into different kingdom/phyla.

Phosphorylation motifs and kinases can be classified as basophilic, acidophilic or proline directed. The logos from the top discriminative n-grams from the animal species are distinctly basic, with arginine residues in a number of positions on both the N- and C-terminal sides of the phosphoserines. In contrast, the logos from the plant species contain a number of acidic residues on the C-terminal side.

There are a large number of basophilic kinases in animals, belonging to several families. Interestingly, two articles analyzing the kinomes of *A. thaliana* and rice did not find any members of the basophilic kinases PKA and PKB (from the AGC family) [1, 2]. It is therefore tempting to speculate that the pattern of basic residues in the logos from the animal species is created by basophilic kinases that are specific to or overrepresented in animals, and that PKA and PKB are among these kinases.

Correspondingly, it is likely that acidophilic kinases in plants contribute to the pattern of C-terminal acidic residues in the plant n-gram logos. The best characterized acidophilic kinase is CK2 [6, 43], but that kinase is found in both plants and animals [44, 45]. This makes it likely that other kinases catalyze the serines in the characteristic logos. Alternatively, CK2s may be more active in plants. Another potential source of distinctive phosphorylations in plants is the receptor like kinase (RLK) family. This large family is unique to plants [46].

The amino acid patterns in the logos from the n-grams specific for fungi are not as distinct as those from plant and animal species. However, particularly in the logos from *S. pombe*, a number of proline residues C-terminal to the phosphoserines can be seen. Proline directed kinases are ubiquitous and more than a quarter of all sites identified in large-scale phosphoproteomics experiments belong to this category [6]. A majority of the well-characterized proline directed kinases (for example CDK1, GSK3 and MAPK3) have a requirement for a proline immediately after the phosphoserines [43, 47]. The logo specific for *S. pombe* has proline residues at position 3–7. A similar, but less pronounced pattern can be seen in the logo for *S. cerevisae*. This opens the possibility that hitherto uncharacterized proline-directed kinases are responsible for the phosphorylation of the sites making up the fungi-specific logos.

The differences between the logos from the different groups of species indicate that there are distinct groups of kinases with dominating activities in the different groups of species. Identifying the kinases responsible for the phylum/kingdom specific phosphorylation patterns, would both be of theoretical interest and open possibilities for practical applications. For example, inhibitors of these kinases could potentially be used as candidates for novel fungicides.

### Secondary structure prediction

Our results of prediction of secondary structure show that for all the species considered here there is a greater proportion of random coil among the serine centered phosphopeptides holding the discriminative n-grams than in the whole protein that they are derived from (Table 5). This means that on average the 88 % of the sequence of the discriminative n-grams holding serine centered 21 mer phosphopeptides do not have a regular secondary structure. Thus they are in disordered regions that can have a rapid evolution due to their lack of structural constraints [4, 5, 48]. The 21-mer serine centered phosphopeptides holding the discriminative n-grams have a lower proportion of random coils than the 13 mer serine centered phosphopeptides (Table 5). There were different proportions of random coils among different species. This lack of structural constraints among the phosphosites and the surrounding residues might explain the hydrophobicity distributional preferences found (Fig. 3 & Additional file 1: Figure S2). The placement of hydrophobic amino acids on the protein surface would form well packed interfaces, in contrast phosphosites tend to have a local decrease of hydrophobic residues and enrichment in surface exposed residues in order to be highly accessible for the kinases and phosphatases [5, 49].

**Table 5:** Proportion of coils in the discriminative n-gram holding proteins and 21 and 13 mer serine centered phosphopeptides among the different species in the training and testing set

Species	Proportion of coils in the proteins	Proportion of coils in the 21 mer phosphopeptides	Proportion of coils in the 13 mer phosphopeptides
*Arabidopsis thaliana*	62 %	85 %	88 %
*Homo sapiens*	67 %	90 %	92 %
*Drosophila melanogaster*	69 %	91 %	94 %
*Saccharomyces cerevisiae*	63 %	86 %	89 %
*Plasmodium falciparum*	71 %	89 %	89 %
*Oryza sativa*	64 %	87 %	92 %
*Mus musculus*	69 %	89 %	92 %
*Caenorhabditis elegans*	63 %	88 %	91 %
*Schizosaccharomyces pombe*	63 %	90 %	92 %
*Toxoplasma gondii*	64 %	80 %	86 %

## Conclusions

Through the generation of classification models and evaluation of discriminative n-grams the evolutionary divergence of protein phosphorylation was studied. The normalized frequencies of the n-grams discriminating the species in the training set were able to classify correct kingdom/phylum for the species in the test set. We also described their properties and identified discriminative motifs that were not selected because of being a consequence of species-specific or kingdom/phylum specific protein inventory.

This analysis provides a framework for the generation of biological insights by comparative analysis of high-throughput phosphoproteomics datasets. We expect the rapidly growing data from high-throughput mass spectrometry analysis will make comparative phosphoproteomics a powerful tool for elucidating the evolutionary changes of reversible phosphorylation that contribute to kingdom/phylum divergence to be applied in several study areas.

## Additional files

Additional file 1: Table S1. Motif overlap detected by Motif-x and MMFPh and by the two serine centred phosphopeptide lengths (13 and 21) in the *Phytophthora infestans* dataset. **Table S3.** *Phytophthora infestans* unique significant motifs and motifs with significantly higher score tested by means of ANOVA. These motifs sorted by the motif score: log (foreground matches/foreground size)/(background matches/background size) and are found to be unique when comparing motifs in *P. infestans*, *A. thaliana*, *S. cerevisiae* and *Homo sapiens* (Additional file 1: Table S1) from both methods and phosphopeptide lengths. **Figure S1.** Clusters of discriminative motifs of equal physico-chemical nature. Clusters are created by grouping motifs according to hydrophobicity, negative, positive and proline content binary values. **Figure S2.** Proportion of hydrophobic (A), negative (B), positive (C) and proline (D) amino acids within each cluster of discriminative N-grams. Additional file 2: Table S2. Alignment of motif scores from *P. infestans*, *A. thaliana*, *S. cerevisiae* and *Homo sapiens.*
Additional file 3: Table S4. Phosphopeptides from the species in the training and testing set with the 5540 discriminative n-grams composing the signature. Additional file 4: Table S5. N-gram derived proteins in each species with their orthologs in other species.
