# Supplementary material for: Comparison of phosphorylation patterns across eukaryotes by discriminative N-gram analysis
Source: BMC Bioinformatics. 2015 Jul 30;16(1):239. doi: 10.1186/s12859-015-0657-2 (PMC4520095; doi:10.1186/s12859-015-0657-2)
Supplement: Additional file 1: Table S1. — Motif overlap detected by Motif-x and MMFPh and by the two serine centred phosphopeptide lengths (13 and 21) in the Phytophthora infestans dataset. Table S3. Phytophthora infestans unique significant motifs and motifs with significantly higher score tested by means of ANOVA. These motifs sorted by the motif score: log (foreground matches/foreground size)/(background matches/background size) and are found to be unique when comparing motifs in P. infestans, A. thaliana, S. cerevisiae and Homo sapiens (Additional file 1: Table S1) from both methods and phosphopeptide lengths. Figure S1. Clusters of discriminative motifs of equal physico-chemical nature. Clusters are created by grouping motifs according to hydrophobicity, negative, positive and proline content binary values. Figure S2. Proportion of hydrophobic (A), negative (B), positive (C) and proline (D) amino acids within each cluster of discriminative N-grams. [file 12859_2015_657_MOESM1_ESM.pdf]

Table S1: Motif overlap detected by Motif-x and MMFPh and by the two serine centred phosphopeptide lengths (13 and 21) in the *Phytophthora infestans* dataset.

**METHODS**	**motifs**
mmfph only	87
motifx only	23
mmfph & motifx	29
Total mmfph	116
Total motifx	52
**21 vs 13-mer serine centred phosphopeptide lengths**	**motifs**
13-mer only	6
21-mer only	23
13 & 21-mer	110
Total 13-mer	116
Total 21-mer	133

Table S3: *Phytophthora infestans* unique significant motifs and motifs with significantly higher score tested by means of ANOVA sorted. These motifs sorted by the motif score: log (foreground matches/foreground size)/(background matches/background size) and are found to be unique when comparing motifs in *P. infestans* , *A. thaliana*, *S. cerevisiae* and *Homo sapiens* (Supplemental table S1) from both methods and phosphopeptide lengths.

Uniquely enriched significant motifs	Score
........P.SPR........	5.576
.......RRGS..........	4.704
..........SP.......P.	3.455
..........SP.....P...	3.434
P.........SP.........	3.361
.......K..SV.........	3.224
.........GSF.........	3.095
P......R..S..........	2.863
.......R..S........P.	2.591
........R.SF.........	2.591
.......R..S......P...	2.578
.......R..S....R.....	2.452
P.......R.S..........	2.412
......K.R.S..........	2.345
..........SF...R.....	2.327
........RGS..........	2.154
.....R...GS..........	2.119
..........S.....RS...	1.857
....KL....S..........	1.820
.....L....SP.........	1.817
......SR.....	1.395
......R...S..........	1.367
..........S.R........	1.304
Motifs with significantly higher score	Score
..........SPR........	3.777
.....R.R..S..........	3.393
.......RR.S..........	3.286
.....L.R..S..........	3.148

Figure S1: Clusters of discriminative motifs of equal biochemical nature. Clusters are created by grouping motifs according to hydrophobicity, negative, positive and proline content binary values.

Cluster1

Homo sapiens	*Mus musculus*
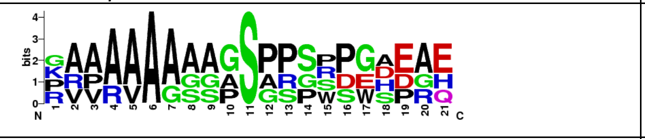	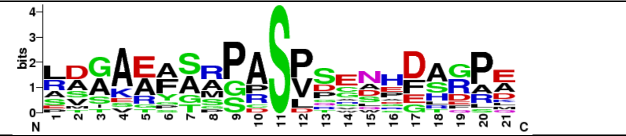
Drosophila melanogaster	*C. elegans*
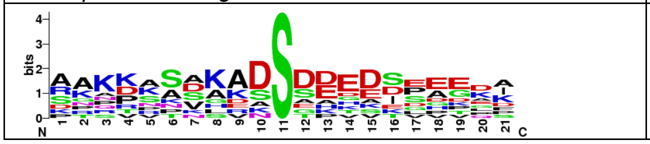	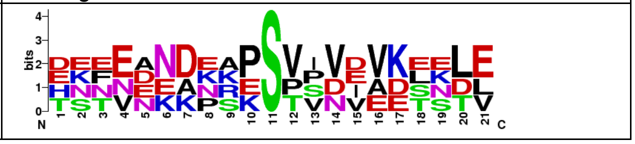

Cluster2

A. thaliana	*Oryza sativa*
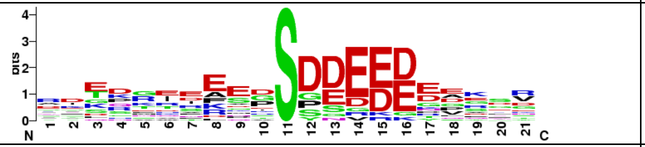	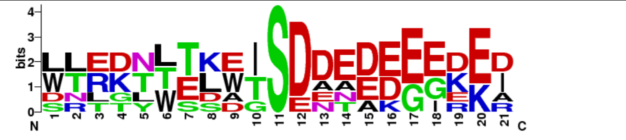
Drosophila melanogaster	*C. elegans*
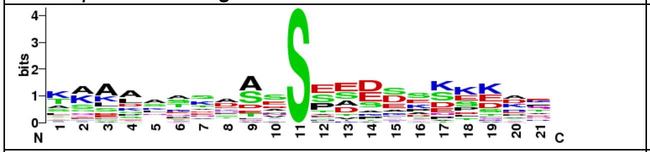	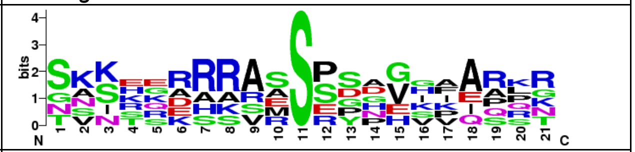
S. cerevisae	S. pombe
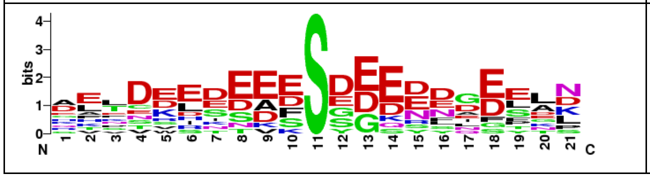	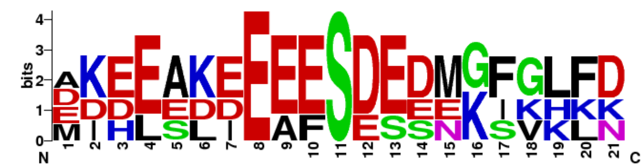

Cluster3

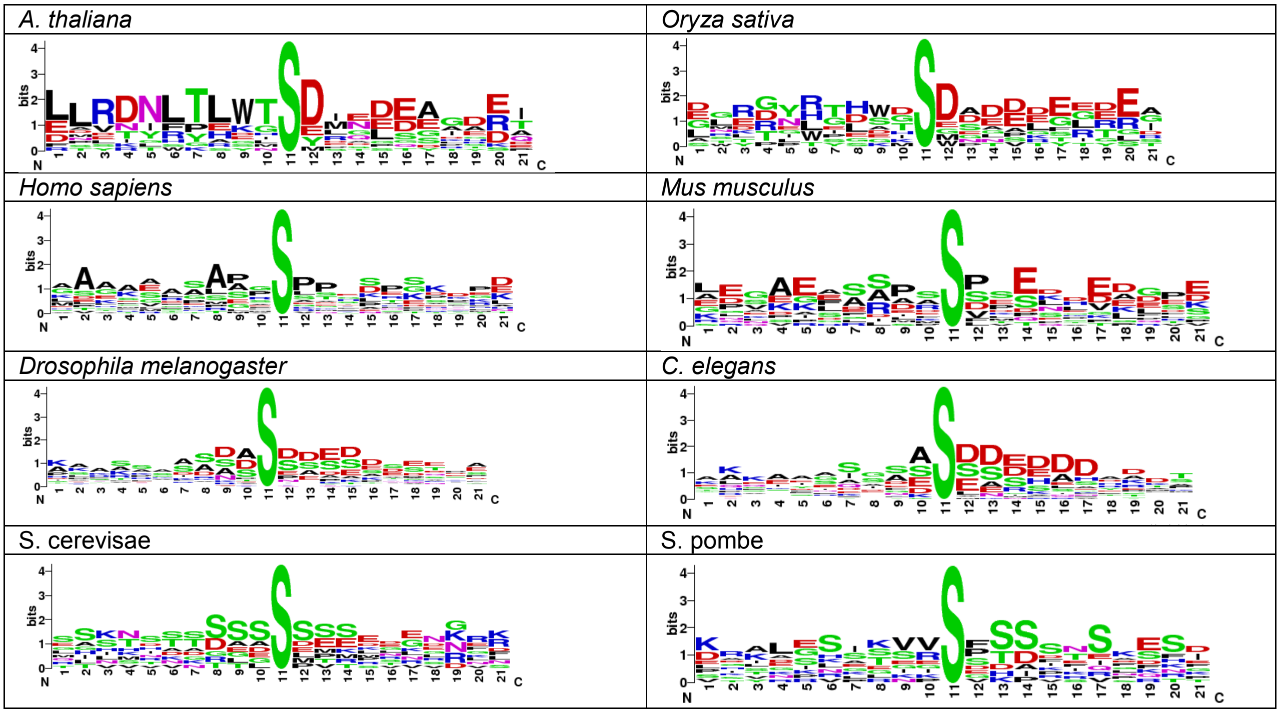

Cluster 4

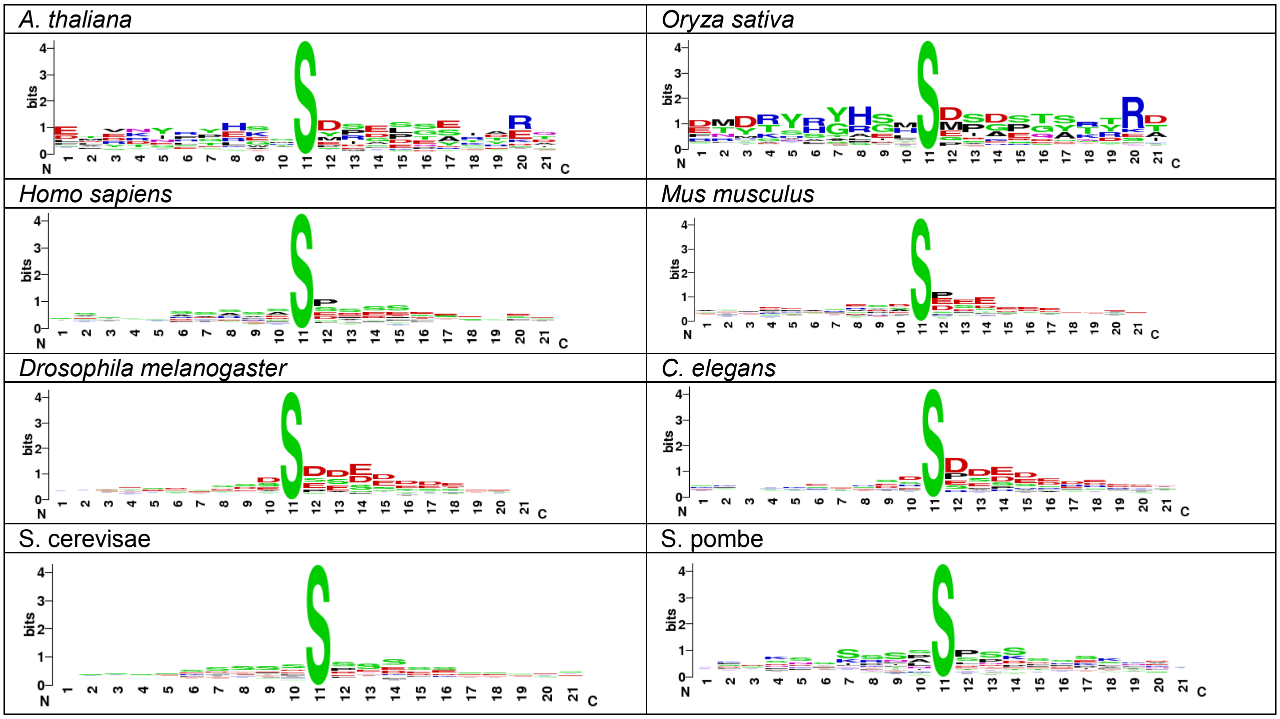

Cluster 5

A. thaliana	*Oryza sativa*
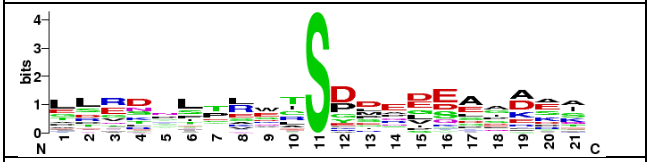	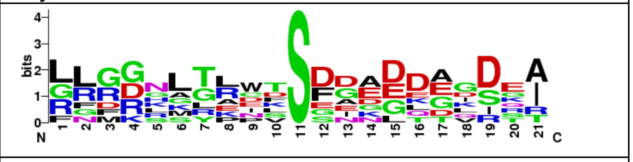
Homo sapiens	*Mus musculus*
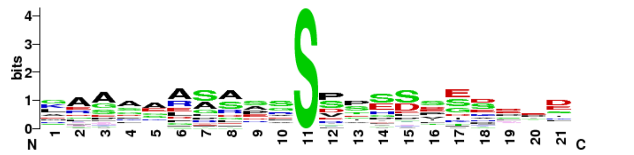	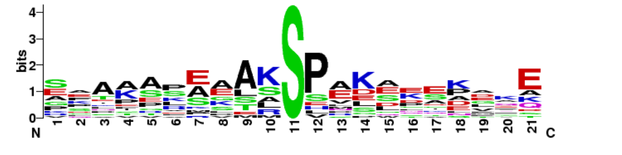
Drosophila melanogaster	*C. elegans*
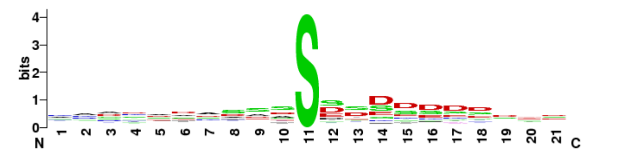	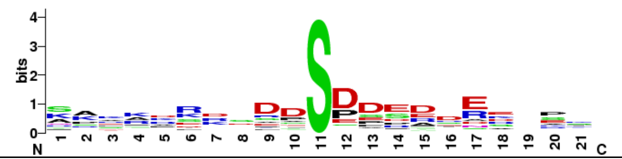
S. cerevisae	S. pombe
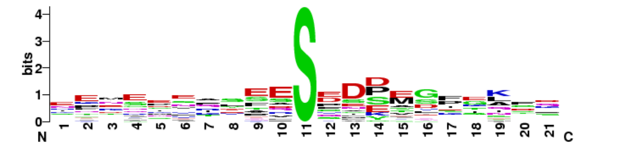	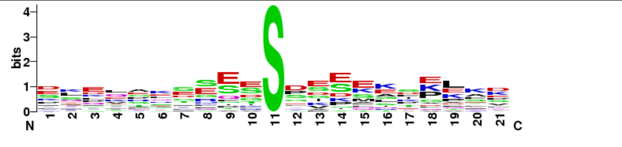

Cluster6

A. thaliana	*Oryza sativa*
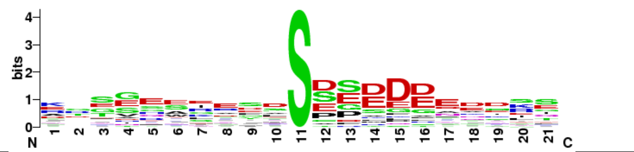	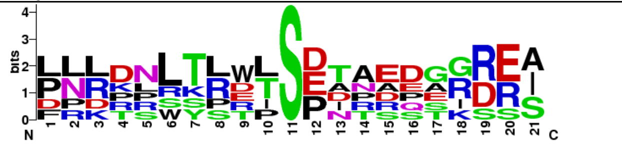
Homo sapiens	*Mus musculus*
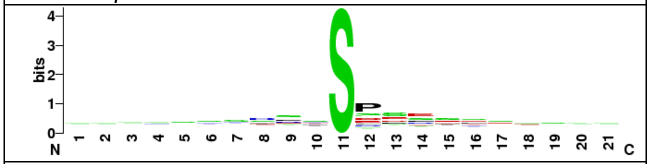	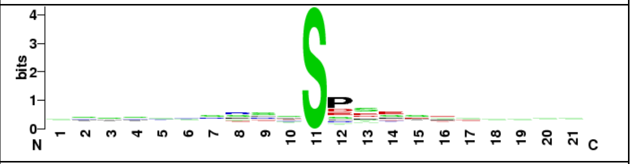
Drosophila melanogaster	*C. elegans*
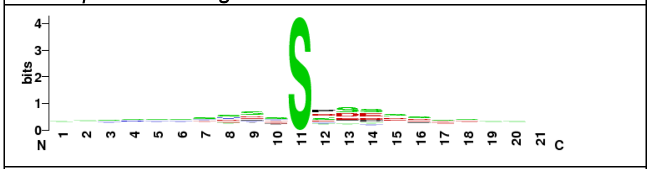	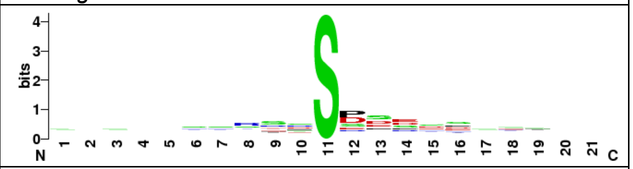
S. cerevisae	S. pombe
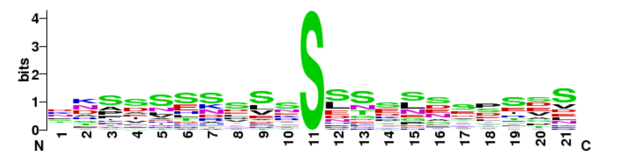	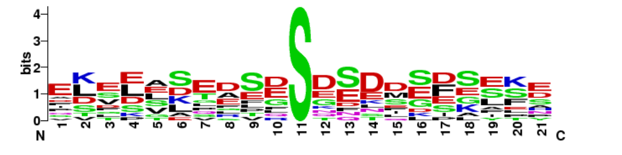

Cluster 7

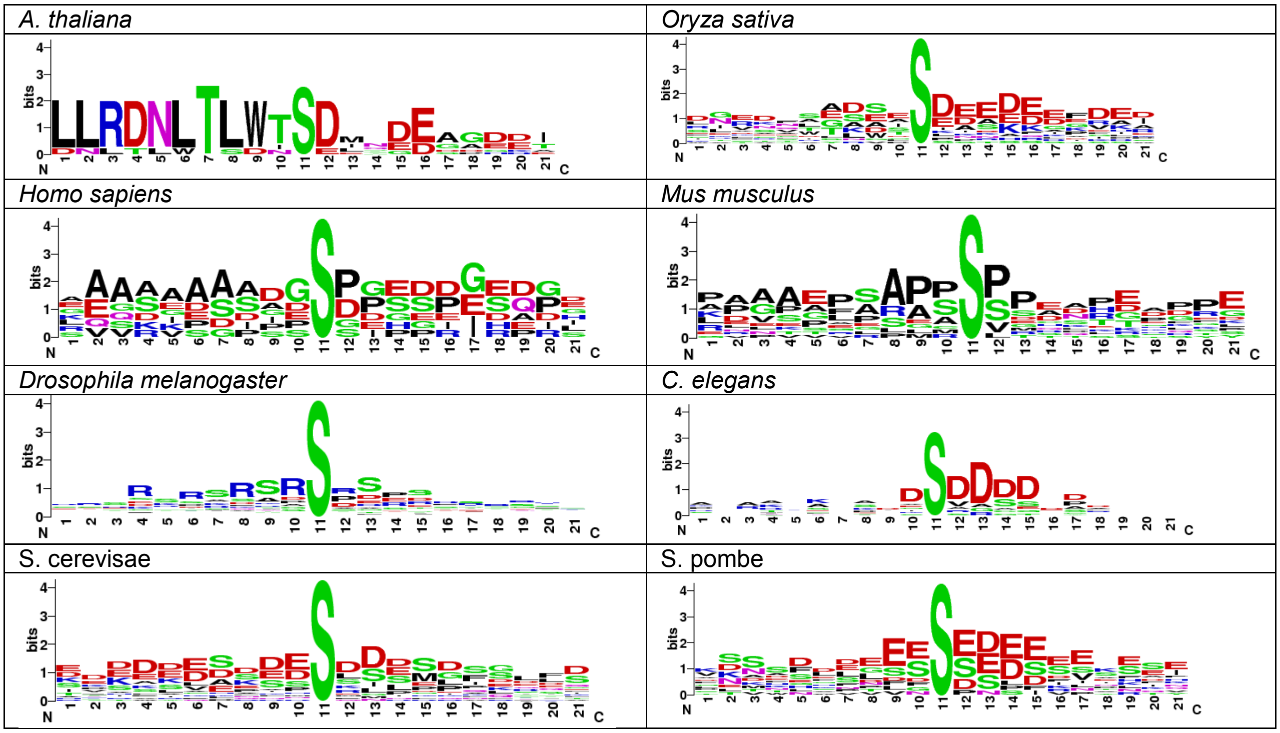

Cluster 8

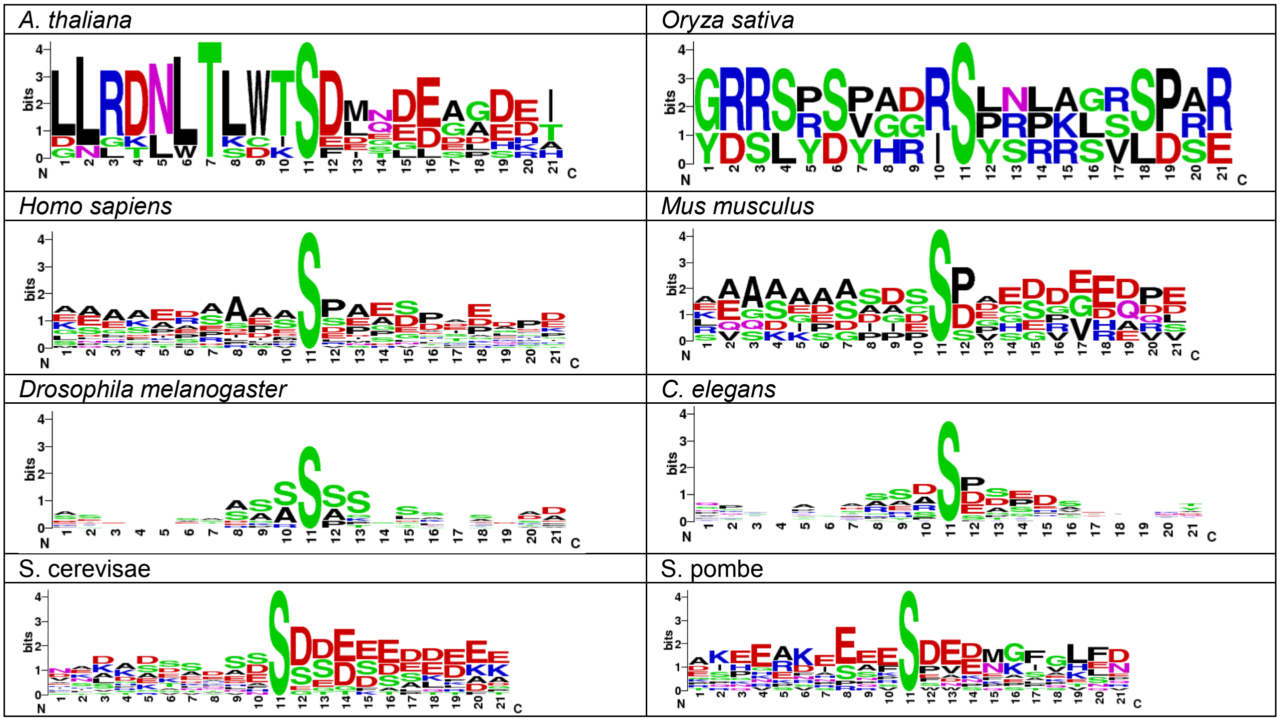

Cluster 9

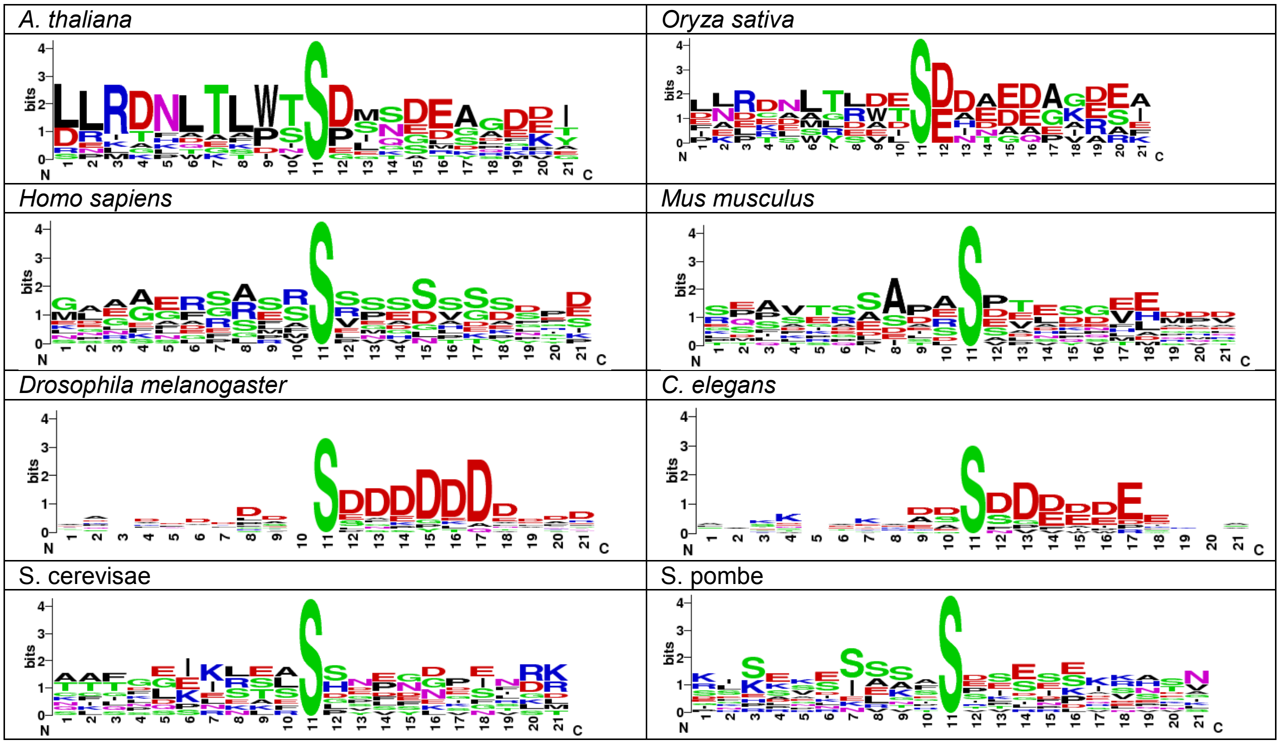

Cluster 10

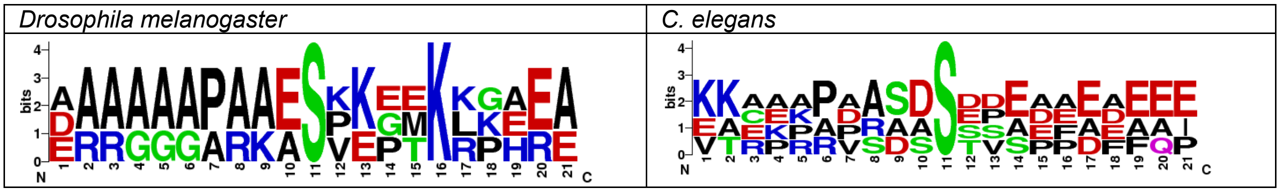

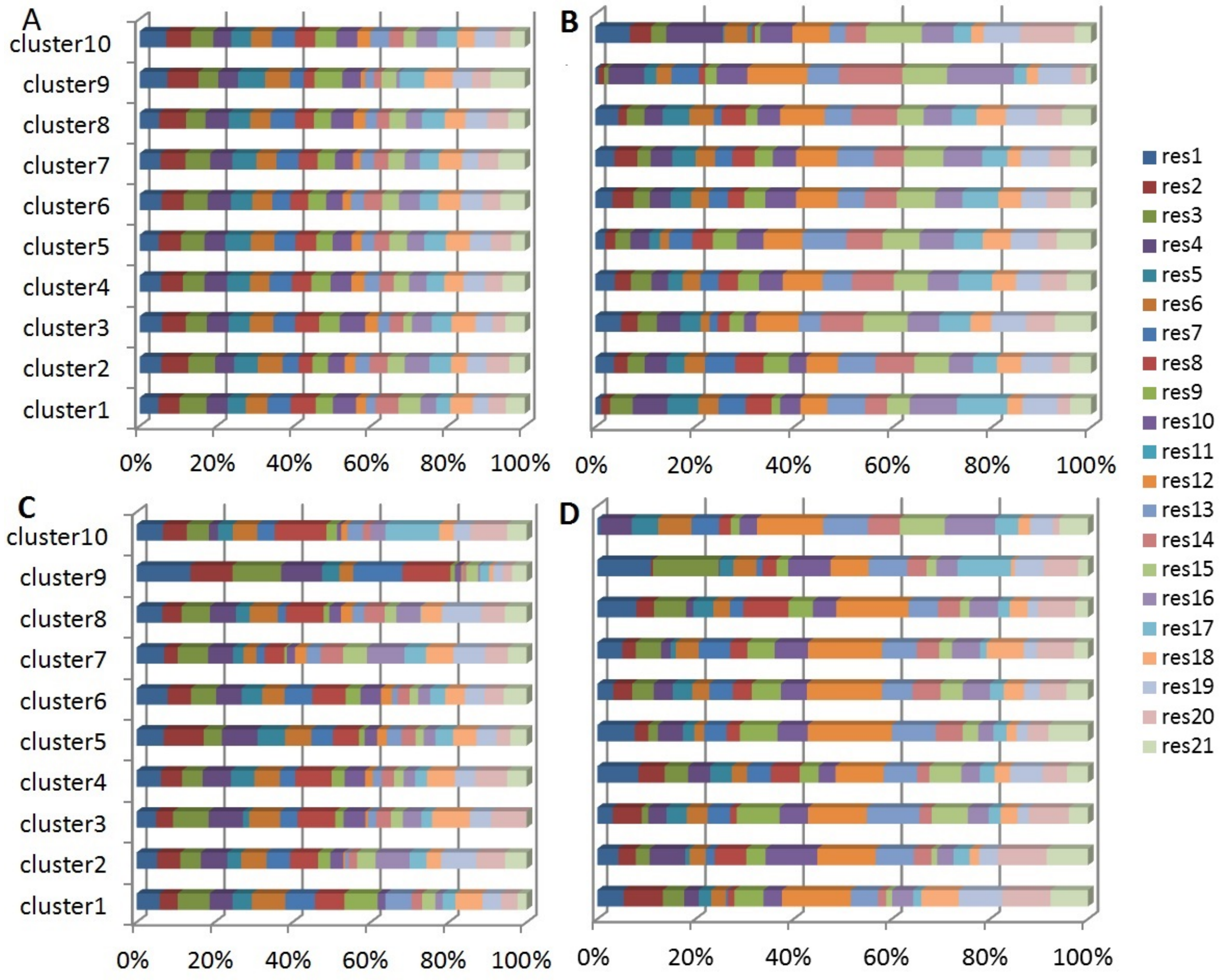

Figure S2: Proportion of hydrophobic (A), negative (B), positive (C) and proline (D) amino acids within each cluster of discriminative N-grams.
